# Prefer cheap or expensive products? Shopping stage matters

**DOI:** 10.3389/fpsyg.2024.1418082

**Published:** 2024-10-09

**Authors:** Wanshu Niu, Wuke Zhang, Mingliang Chen, Minghua Han

**Affiliations:** ^1^School of Business, Ningbo University, Zhejiang, China; ^2^School of Management, Zhejiang University, Hangzhou, China

**Keywords:** product price, virtual shopping cart, shopping stages, perceived product quality, perceived monetary sacrifice

## Abstract

E-commerce platforms generally provide consumers with the function of virtual shopping carts to help them store interested products. Although about 80% of online consumers intends to purchase after adding products to their shopping cart, the abandonment rate of cart products has been up to 70%. It is important to understand how to improve consumer attitude toward product both before and after cart use. Building on the relevant literature and the consumer shopping goal stages theory, this study aims to examine the effects of product price, as one of the most indispensable and important information in online shopping, on consumer attitudes toward product at different decision-making stages in online shopping (i.e., add-to-cart stage and place-an-order stage), and the mediating roles of perceived product quality and monetary sacrifice. The findings from behavioral experiment suggest that high price leads to a more positive attitude toward product at add-to-cart stage by strengthened perception of high product quality, while results in a less positive product attitude at place-an-order stage because of the enhanced perception of monetary sacrifice. Both theoretical contributions and practical implications are discussed.

## Introduction

Product price is an indispensable piece of information in online shopping. For consumers, price is not only the monetary sacrifice they should pay to obtain the product, but also a useful indicator of product quality (Rao, 2005; Shirai, 2015; Wang and Chen, 2016). With the increasingly fierce market competition in e-commerce, many e-retailers utilize lower product prices to attract consumers to purchase. However, without considering consumer perception at different decision-making stages, a low-price strategy may not only waste marketing budgets, but also lose the attractiveness of products to consumers. For example, low product prices may result in low quality perception and, thus, be excluded from consumers’ consideration list, while high product prices may make consumers give up purchases due to their limited budget. Although the extant literature has examined the impact of factors such as product features (Shirai, 2015; Völckner et al., 2012) and consumer characteristics (Park et al., 2020; Septianto et al., 2021; Yang et al., 2019) on different perceptions of price, most previous studies have focused on the impact of price on consumers’ final purchases, and there is still a lack of understanding of how to utilize price information to attract consumers at different decision-making stages.

Previous research suggest that add-to-cart and place-an-order are two key decision-making stages in online shopping (Yang and Lai, 2006; Kukar-Kinney and Close, 2010; Moriguchi et al., 2016). Add-to-cart is the stage in which consumers select products of interest to add to the shopping cart, whereas place-an-order is the stage in which consumers place an order for products on the cart list (Close and Kukar-Kinney, 2010). Understanding consumers’ attitudes toward products in the add-to-cart and place-an-order stages is of great importance. The convenience of online shopping makes it easier to suspend or abandon the decision-making process at any time (Summer, 2021). It has been reported that approximately 80% of online consumers intend to purchase after adding products to their shopping cart (Sabanoglu, 2020), and the rate of cart product abandonment is as high as 70% (Coppola, 2020). Thus, promoting consumer attitudes toward products at each decision-making stage is essential for e-retailors to facilitate final purchases and maximize profits. On the other hand, with the development and maturity of online shopping cart-tracking technologies, the stages of add-to-cart can be clearly distinguished from the stage of place-an-order, consumers’ behaviors at each stage can be accurately recorded, and the accessible product information can be easily manipulated to implement marketing strategies (Li et al., 2020; Luo et al., 2019). However, to date, little attention has been paid to exploring how price information is perceived and induces product attitudes at different decision-making stages (i.e., add-to-cart and place-an-order stages).

To fill this gap in the literature, in light of the consumer shopping goal stage theory, our study aims to examine the effects of price on consumer attitudes toward products at different decision-making stages (i.e., add-to-cart and place-an-order) and the mediating roles of perceived quality and monetary sacrifice. A controlled behavioral experiment was conducted to test the proposed hypotheses. Our findings not only contribute to the extant knowledge on price, virtual shopping carts, and the application of shopping goal stage theory but also provide practical implications for price-targeting marketing strategies at the add-to-cart and place-an-order stages.

In the following, we first review the literature on the two decision-making stages of add-to-cart and place-an-order, the dual roles of product price, and the consumer shopping goal stage theory. Section 3 proposes a theoretical research model and develops the four hypotheses. Section 4 and Section 5 elaborates on our experiment and the hypotheses’ testing results. Finally, we discuss the theoretical contributions and practical implications of the findings.

## Theoretical background

### Two decision stages in the online shopping context

E-commerce platforms generally provide consumers with virtual shopping carts. Similar to physical purchase scenarios, a virtual shopping cart provides online consumers with a virtual space to store products they are interested in during their shopping process (Close and Kukar-Kinney, 2010). Cart usage naturally distinguishes between two stages in the consumer decision-making process: add-to-cart and place-an-order. The add-to-cart stage refers to the stage in which the product is added to the carts, whereas the place-an-order stage refers to the stage in which products are selected from the cart list to place an order (Kukar-Kinney and Close, 2010; Close and Kukar-Kinney, 2010). Scholars have clarified the theoretical distinctions between add-to-cart and place-an-order stages. For example, Kukar-Kinney and Close (2010) proposed that consumers form their consideration set of products at the add-to-cart stage and make final purchase decisions at the place-an-order stage. Luo, Lu (Luo et al., 2019) utilized the theory of shopping goal stages and proposed that consumers form their shopping goals at the add-to-cart stage and realize them at the place-an-order stage.

Most previous studies have focused on exploring the factors influencing shopping cart usage at the add-to-cart stage, and shopping cart abandonment at the place-an-order stage. For example, the literature suggests that factors related to shopping motivation (Close and Kukar-Kinney, 2010; Boyle et al., 2018) and risk perceptions (Cho et al., 2006) could significantly influence consumers’ intention to add products to carts, whereas factors related to uncertainty (Tang and Lin, 2019; Huang et al., 2018) and risk perceptions (Moore and Mathews, 2008; Rajamma et al., 2009) are likely to affect whether consumers abandon products in the cart list at the place-an-order stage. In fact, factors (e.g., low levels of perceived uncertainty and risk) that lead consumers to engage in the add-to-cart stage may not be sufficient to place an order, or vice versa. For example, although the perception of product description uncertainty has been proven to affect cart usage (Jiang and Benbasat, 2004), it does not significantly impact consumer behavior at the place-an-order stage (Tang and Lin, 2019).

While extant studies on shopping carts have widely explored factors at the add-to-cart or place-an-order stages, we notice that there is still a lack of direct comparisons of the differences in the influence of the same factor at different stages. Additionally, previous studies have allocated little effort to factors related to product information in either the add-to-cart or place-an-order stages. Considering the indispensability and importance of product prices at all decision-making stages, it is worth exploring how the same piece of price information affects consumers’ attitudes toward products in the add-to-cart and place-an-order stages.

### Dual roles of product price

A large number of studies have verified that product price plays an important role in consumer product evaluation (Boyle et al., 2018; Liu et al., 2020; Somervuori and Ravaja, 2013; Umashankar et al., 2017; Wilkins and Ireland, 2020). On the one hand, according to classical economic theory, price is an indicator of the economic cost of purchasing a commodity. A higher price means that consumers need to pay more monetary sacrifices, resulting in a negative correlation between the price level and purchase probability. On the other hand, consumers rely on price information to infer product quality. The so-called you get what you pay for indicates that higher prices can increase consumers’ perception of product quality and thus have a positive impact on purchasing probability (Rao, 2005; Chang, 2013).

The literature has extensively explored the factors that lead consumers to perceive prices more as indicators of product quality or monetary sacrifice. Some scholars have argued that product-related factors significantly influence price perceptions. For example, Völckner, Rühle (Völckner et al., 2012) found that when products are partition priced, consumers are more inclined to make quality judgments, instead of focusing on monetary sacrifice perceptions. Moreover, consumers’ impulsivity (Kukar-Kinney et al., 2012), fluency in processing information (Chang, 2013), type of self-identity (Yang et al., 2019), and psychological traits (Bornemann and Homburg, 2011) have also been explored in relation to quality perceptions of price. For example, Bornemann and Homburg (Bornemann and Homburg, 2011) show that when psychological distance is relatively distal, consumers are more inclined to make quality judgments based on price, whereas when psychological distance is closer, consumers tend to focus on monetary sacrifice perceptions induced by price.

Although extant literature has investigated the impact of various factors, such as product features and consumer characteristics, on different perceptions of price, most of them focus on the impact of price on consumers’ final purchases, ignoring the possible influence of decision-making stages. A series of prior studies have consistently proven that consumers may utilize different information and exist different psychological perceptions in different decision-making stages (Levin et al., 2005; Hubl and Trifts, 2000; Guo et al., 2012). Thus, it is worth exploring how consumers perceive price information and develop attitudes toward products at different decision-making stages.

### Consumer shopping goal stage theory

The consumer shopping goal stage theory was proposed by Lee and Ariely (2006) to explain how the influences of shopping goal concreteness and external factors differ between the two stages of consumers’ decision-making processes. In the initial stage, consumers generally browse, collect information, and compare products, because they have not yet constructed concrete shopping goals. Hence, consumers have a deliberative mind-set in the first stage, where they are more open-minded, easily influenced by external factors, and strive to establish a concrete goal (Fujita et al., 2007). When consumers have already constructed a specific shopping goal, they enter the second stage and strive to achieve the goal. Hence, consumers tend to have an implemental mindset in the second stage, where they are more dedicated to the goal and less likely to be influenced by contextual factors unrelated to the goal (Gollwitzer and Bayer, 1999; Gollwitzer et al., 1990; Gagné and Lydon, 2001).

Previous studies have utilized shopping goal stage theory to investigate how different information should be targeted to consumers at different stages to improve their attitude toward a product (Luo et al., 2019; Song et al., 2017; Trzebinski et al., 2023; Chandran and Morwitz, 2005). For example, Luo, Lu (Luo et al., 2019) applied this theory to theorize add-to-cart as the first stage and place an order as the second stage. The results of the field experiments showed that advertisement targeting can help consumers in the second stage (i.e., place-an-order stage) identify products consistent with their shopping goals. Thus, targeting advertising is more effective in the place-an-order stage than it is in the add-to-cart stage. Additionally, the promotional effect of targeted advertising is further enhanced when the advertisement includes information on price promotions or prompts for scarce product supply (e.g., “the product only has 2 items left”).

As researchers have suggested, targeting different types of information at specific stages has a critical impact on consumers’ attitudes toward a product. However, very limited effort has been devoted to leveraging this theory to investigate how the same piece of product information leads to different consumer attitudes toward products at different stages. In practice, most product information, such as price, on e-commerce platforms is open to consumers at all stages of decision making. Given that consumers at different stages have different mindsets, it is worth exploring whether the same information can have different impacts on consumer perceptions and product attitudes at different stages. Considering price as one of the most indispensable and important pieces of product information, we propose a research model to examine the effects of price on consumers’ attitudes toward products at different stages (i.e., add-to-cart and place-an-order stages), as well as the mediating roles of perceptions of product quality and monetary sacrifice.

## Hypothesis development

As we have mentioned, consumers tend to perceive high product prices as indicators of high product quality and monetary sacrifices. From a consumer’s perspective, obtaining high-quality products is undoubtedly an ideal shopping goal. However, considering the affordability of financial burdens, the high monetary sacrifice brought about by high-priced products often forces consumers to make feasible judgments (Bornemann and Homburg, 2011; Yan and Sengupta, 2011). According to Lee and Ariely (Lee and Ariely, 2006) shopping goal stage theory, consumers in the first stage (i.e., add-to-cart) are uncertain about their shopping goals. Thus, they have a deliberative mindset and are committed to excluding those obviously undesirable products to shorten product lists (Luo et al., 2019; Block and Morwitz, 1999). When shopping on the Internet, consumers cannot directly examine the actual quality and performance of products, which leads to a strong dependence on price perceptions (Rao, 2005). In light of this, although products with high monetary sacrifices may not be strongly desired, low-quality products are highly likely to be categorized as completely unwanted ones. To shorten product lists in the add-to-cart stage, consumers should emphasize quality perception over cost perception. Thus, we contend that consumers at the add-to-cart stage are likely to depend more on price information.

By contrast, consumers in the second stage (i.e., place-an-order) have already constructed a concrete shopping goal and strive to achieve it. Thus, they have an implemental mindset and allocate more efforts on how to get the desired product (Lee and Ariely, 2006; Brandstatter and Frank, 2002). Price information at the place-an-order stage is directly related to the monetary sacrifice consumers should pay to obtain the product. Although high-quality products are ideal choices, consumers should consider their budgets and may have to give up some of the desired products when focusing on “how” to place an order. Echoing this, prior research has also argued that consumers are more likely to perceive price as an indicator of monetary sacrifice at the place-an-order stage because lower costs can effectively help them identify desired products (Luo et al., 2019; Wakefield and Bennett, 2014; Hustic and Gregurec, 2015). Hence, we contend that consumers in the place-an-order stage tend to rely on price to infer costs more than quality to facilitate the implementation of the final purchase. Thus, we propose:

*H1: The positive effect of price on consumers’ perception of product quality is stronger at add-to-cart stage (vs. place-an-order stage).*

*H2: The positive effect of price on consumers’ perception of monetary sacrifice is stronger at place-an-order stage (vs. add-to-cart stage).*

Consumers’ target action at the add-to-cart stage involves adding products to shopping carts, whereas the target action at the place-an-order stage is the actual purchase of products. Considering the potential impact of different target actions on our hypothesized effects, our study focuses only on consumers’ attitudes toward a product as a dependent variable. On the one hand, previous studies have generally agreed that when consumers pay attention to the quality information conveyed by prices, the higher quality perception brought by higher prices leads to a more positive product attitude (Dodds et al., 1991; Erickson and Johansson, 1985). In other words, perception of product quality should mediate the effects of price on product attitudes. As we propose that the positive effect of price on quality perception is stronger at the add-to-cart stage, we contend that price leads to a more positive attitude toward a product because of its stronger effect on perceived quality.

**Figure 1:**
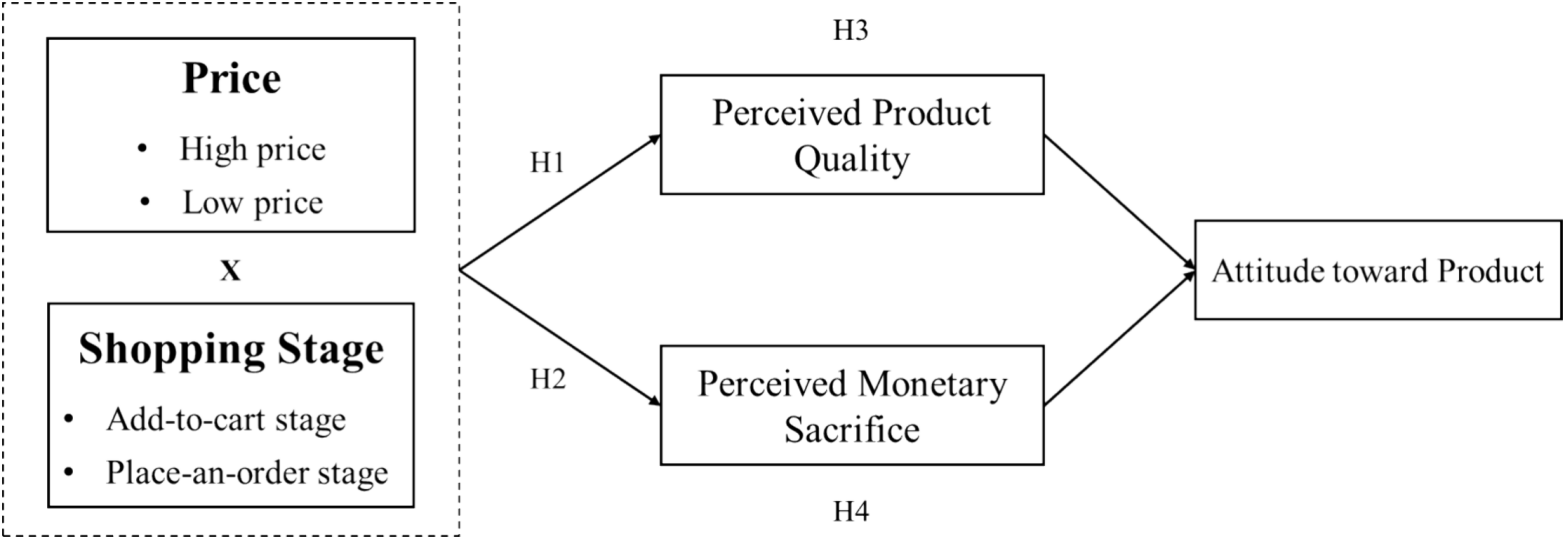
Conceptual model.

On the other hand, there is also evidence that when consumers pay attention to the cost information conveyed by prices, the increasing perception of monetary sacrifices will decrease their product attitudes and purchase intentions (Dodds et al., 1991). In other words, the perception of monetary sacrifices should mediate the effects of price on product attitudes. As we propose that the positive effect of price on perceived monetary sacrifice is stronger at the place-an-order stage, we contend that price leads to a less positive attitude toward a product because of its stronger effect on perceived monetary sacrifice (Figure 1). Thus, we propose:

*H3: Consumers’ attitude toward product at add-to-cart stage (vs. at place-an-order stage) is more positive because of the strengthened effect of price on perception of product quality.*

*H4: Consumers’ attitude toward product at place-an-order stage (vs. at add-to-cart stage) is less positive because of the strengthened effect of price on perception of monetary sacrifice.*

## Methods

We followed three steps to empirically test the four hypotheses. First, we conducted a pre-test experiment to determine the manipulation of product prices. Second, a controlled behavioral experiment was conducted with college students from universities in Southeastern China. Third, we performed data analysis with manipulation checks and hypotheses testing.

### Stimuli and manipulation

We adopted a 2 (decision stage: add-to-cart stage vs. place-an-order stage) * 2 (product price: high vs. low) between-subject design in the main experiment. Backpacks were chosen as the target product in the experimental scenario for the following reasons: (1) backpacks are equally attractive for both male and female; (2) backpacks are common products in daily lives and are widely used by college students; and (3) backpacks are widely used as target products in IS and Marketing experimental studies (Yan and Sengupta, 2011; Estelami et al., 2006; Page and Herd, 2010; Penttinen et al., 2022). Thus, a pretest experiment was conducted to (1) determine the high and low prices of backpacks for manipulation in the main experiment, and (2) test the attractiveness of the product image to participants of different genders to exclude the potential impact of product image attractiveness.

**Figure 2:**
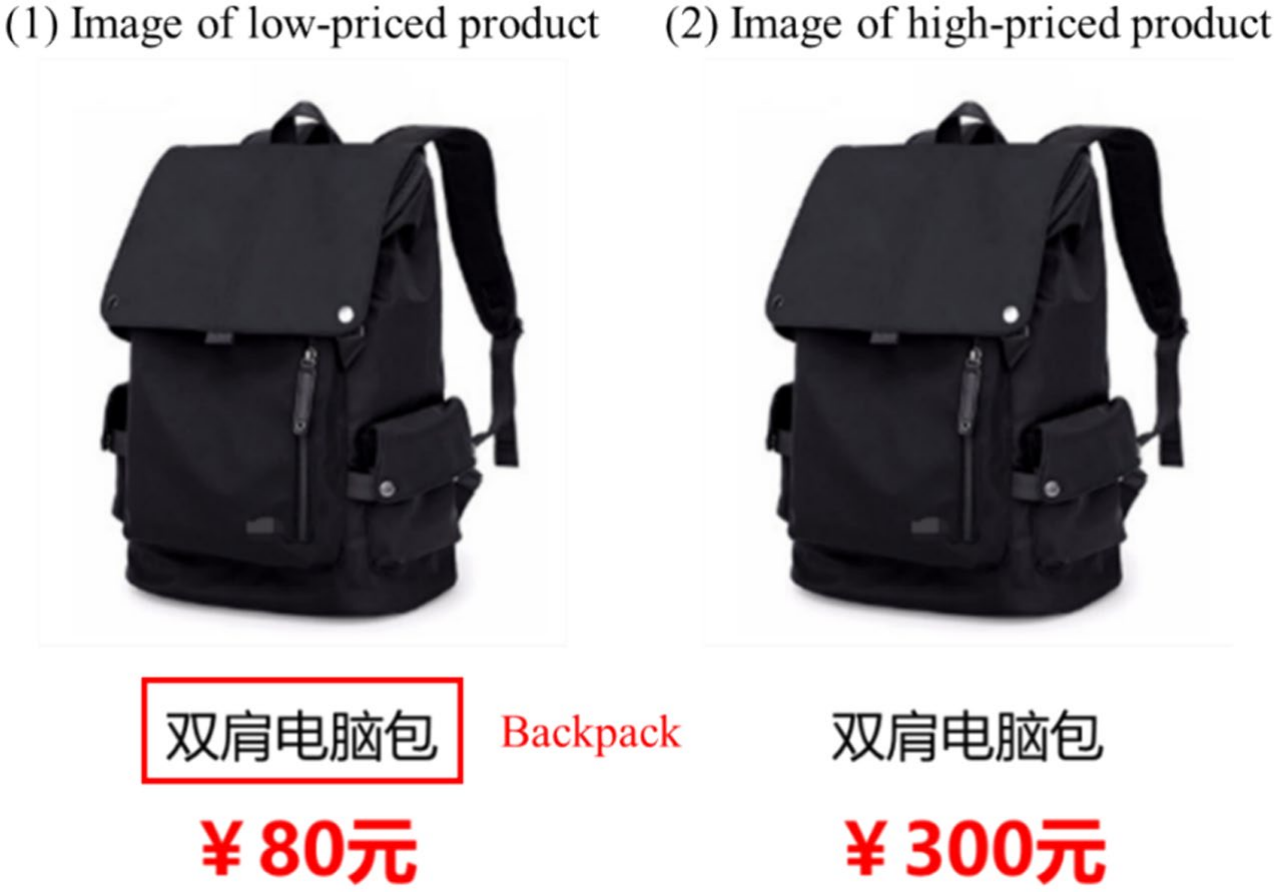
Experimental stimuli design.

The pretest experiment recruited 40 college students with an average age of 23.15 (SD = 2.25), including 18 females (45.00%). To avoid learning effects, participants in the pre-test experiment did not participate in the subsequent main experiment. In the pre-test experiment, participants were asked to imagine a scenario in which they selected backpacks on an online shopping platform. They were then presented with a backpack image and asked to rate the degree to which the backpack image attracted them (not at all/very attractive, 7-point scale). The participants also rated their usage experiences (not at all/very experienced, 7-point scale) and knowledge (not at all/very knowledgeable, 7-point scale) about backpacks and reported the ceiling and floor of the price of the presented backpack that they thought to be reasonable. Finally, participants reported their demographic information such as gender and age and completed the pre-test experiment. According to the results, there was no significant difference in the attractiveness of the backpack images selected in this study among participants of different genders, and it was not significantly related to the participants’ usage experience and knowledge of the backpack. Based on the results of the pretest, this study finally selected RMB 300 yuan as the high price for the backpack and RMB 80 yuan as the low price in the main experiment (as shown in Figure 2). The pretest results are shown in the Table 1.

**Table 1:** Results of pretest.

		Backpack image attractiveness	Low price	High price
Mean (SD)		4.30 (SD = 1.40)	88.42 (SD = 58.92)	308.45 (SD = 212.15)
Gender	Male	4.36 (SD = 1.50)	94.09 (SD = 46.46)	292.27 (SD = 201.14)
	Female	4.22 (SD = 1.31)	83.06 (SD = 74.74)	333.33 (SD = 238.87)
	T-Test	T = 0.31, *p* = 0.76	T = 0.57, *p* = 0.57	T = −0.59, *p* = 0.56
Experience (OLS regression)		β = −0.52, *p* = 0.13	β = 4.49, *p* = 0.77	β = −29.70, *p* = 0.58
Knowledge (OLS regression)		β = 0.39, *p* = 0.17	β = 0.87, *p* = 0.94	β = 6.85, *p* = 0.87

We divided the decision-making process into two stages: (1) add-to-cart stage, in which consumers select products from the product list to add to the shopping cart, and (2) place-an-order stage, in which consumers select products from the shopping cart list to place an order. To manipulate decision stages, participants in all conditions were asked to imagine the following scenario: “You open an e-commerce website app and want to select a product. The purchasing process can be divided into two stages. The first stage involved adding the desired products from the product list to the shopping cart. In the second stage, you choose the final product from the shopping cart and place an order.” Then, for the participants in the “add-to-cart” groups, the main task introduced to them is: “You are currently adding desired product to the shopping cart from a product list, and will not place an order temporarily.” For participants in the “place-an-order” groups, the main task introduced to them is: The first stage of the shopping task was completed. Your current task is to select the desired product from the shopping cart list and place an order directly.

### Subjects and experimental procedure

The main experiment recruited 345 college students from universities in southeastern China with an average age of 23.10, including 166 females (48.12%). All participants voluntarily participated in the experiment. Participants were randomly assigned to one of the four groups: 2 (decision stage: add-to-cart stage vs. place-an-order stage) * 2 (product price: high and low). Firstly, the participants were informed of an online shopping scenario and asked to report their general attitude toward backpacks (“I think backpacks are a great product,” strongly disagree/strongly agree, 7-point scale). Subsequently, participants were presented with scenario descriptions that correspondingly manipulated the decision stages. To test whether the manipulation in the decision-making stage was successful after the scenario description was completed, we used two question items as manipulation checks, “which stage are you currently in?” (“adding items from product list to the shopping cart” or “placing an order directly from the shopping cart list”) and “what actions could you take on the product in current stage?” (“add-to-cart” or “place-an-order”).

Next, all participants read information about the backpack product, including the front image and price of the backpack. All participants rated the perceived product quality, perceived monetary sacrifice, and attitude toward the product (see in Table 2). All items were reflective and measured on a 7-point scale, ranging from 1 (i.e., totally disagree) to 7 (i.e., totally agree). Finally, several control variables were rated, including perceived attractiveness of the product image (not at all/very attractive, 7-point scale), usage experience of backpacks (not at all/very experienced, 7-point scale), familiarity with backpacks (not at all/very familiar, 7-point scale), knowledge of selecting backpacks (not at all/very knowledgeable, 7-point scale), involvement in the experiment (see in Table 2), and current mood (see in Table 2). After reporting demographic information, such as gender and age, all participants received a reward of RMB 4 yuan and were debriefed.

**Table 2:** Measurement items.

Constructs	Items		Reference
Perceived product quality	PQ1	The product appears to be of good quality	Bornemann and Homburg (2011)
	PQ2	The product appears to be reliable	
	PQ3	The product appears to be of high quality	
	PQ4	The product quality appears to be trustworthy	
Perceived monetary sacrifice	EXP1	The product is very expensive	Bornemann and Homburg (2011) and Yan and Sengupta (2011)
	EXP2	The price is very high	
	EXP3	The product requires a lot of money to buy	
Attitude toward product	ATT1	The product is very good	Drolet and Aaker (2002)
	ATT2	The product is very likable	
	ATT3	The product is very favorable	
Involvement	INV1	I am very serious when answering the questions	Yan and Sengupta (2011)
	INV2	I am very involved when answering the questions	
	INV3	I am very thoughtful when answering the questions	
Mood	MOOD1	I am happy	Irmak et al. (2013)
	MOOD2	I am in a very good mood	
	MOOD3	I am very energetic	
	MOOD4	I am very involved	

## Results

### Psychometric properties

First, reliability and validity analyses were conducted using SPSS 24.0. The results showed that the *α* values of the five constructs were all greater than 0.70, indicating good reliability of the construct (Nuimally, 1978). Table 3 shows that the factor loadings of all constructs exceeded 0.71, indicating excellent aggregation validity (Comrey, 1973). In addition, the factor loading of each construct item on that construct was higher than that of the other four constructs, indicating good discriminant validity (Cook et al., 1979; see in Table 3). Therefore, all the five constructs in the main experiment showed good reliability and validity.

**Table 3:** Psychometric properties.

		Cronbach’s alpha	Factor loading				
			PQ	EXP	ATT	INV	MOOD
Perceived product quality	PQ1	0.969	0.866	0.258	0.296	0.002	0.027
	PQ2		0.867	0.285	0.312	−0.008	0.021
	PQ3		0.844	0.283	0.321	0.015	0.026
	PQ4		0.861	0.284	0.313	−0.031	0.051
Perceived monetary sacrifice	EXP1	0.979	0.278	0.940	0.071	0.032	−0.029
	EXP2		0.296	0.937	0.081	0.021	−0.022
	EXP3		0.270	0.926	0.096	0.012	−0.004
Attitude toward product	ATT1	0.922	0.305	0.045	0.890	0.024	0.032
	ATT2		0.468	0.199	0.769	0.000	0.015
	ATT3		0.364	0.049	0.868	−0.015	0.006
Involvement	INV1	0.916	−0.010	0.009	0.045	0.935	0.029
	INV2		−0.003	0.020	−0.026	0.935	0.004
	INV3		−0.001	0.023	−0.010	0.906	0.042
Mood	MOOD1	0.793	0.063	−0.032	−0.032	−0.003	0.838
	MOOD2		0.066	−0.049	0.003	0.033	0.792
	MOOD3		−0.004	0.011	0.004	0.086	0.802
	MOOD4		−0.040	0.028	0.067	−0.039	0.749

### Manipulation check

Two items (that is, ‘Which stage are you currently in?’ and “what actions could you take on the product in current stage?”) indicated that 162 participants in the “add-to-cart” groups answered “adding items from product list to the shopping cart” in the first item and “add-to-cart” in the second item, while 160 participants in the “place-an-order” group answered “placing an order directly from the shopping cart list” in the first item and “place-an-order” in the second item. Therefore, 322 participants were successfully manipulated. Twenty three participants failed and were excluded from the subsequent data analysis.

Following Bornemann and Homburg (30, Study 1), we used the item EXP2 (“The price of the product is very high”) as a manipulation check. Results showed that participants in the high price group perceived a significantly [*F*(1, 320) = 429.27, *p* = 0.00] higher price (Mean = 4.20, SD = 1.23) than those in the low price group (Mean = 1.70, SD = 0.92). Therefore, the experimental manipulation of product prices was successful.

In addition, we conducted ANOVA analysis on the general attitudes toward backpacks of different groups of participants before the hypothesis test. The results showed no significant differences in the general attitude toward backpacks among the four groups of participants. The decision stage [*F*(1, 318) = 0.61, *p* = 0.43] and product price [*F*(1, 318) = 0.89, *p* = 0.35] did not have a significant effect on general attitude toward backpacks. The interaction effect was not significant [*F*(1, 318) = 0.16, *p* = 0.69]. Therefore, we excluded the possible explanation that the differences in the perception of price information and product attitudes reported by the four groups of participants were due to their inherent attitudes toward backpacks.

### Hypotheses tests

#### Effects on perceived product quality and monetary sacrifice

A MANOVA analysis was conducted with price and decision-making stage as independent variables, and perceived product quality and perceived monetary sacrifice as dependent variables (see in Table 4). The results showed no significant effect of decision-making stage on perceived product quality [*F*(1, 318) = 2.92, *p* = 0.09]. The main effect of product price was significant [*F*(1, 318) = 250.26, *p* = 0.00]. Compared with the low price (Mean = 3.15, SD = 1.12), the product with high price (Mean = 4.94, SD = 0.92) was perceived to be of higher quality. Moreover, the interaction effect between the decision-making stage and price was significant [*F*(1, 318) = 7.37, *p* = 0.01]. When participants were at the place-an-order stage, the perceived quality of high-priced (Mean = 4.89, SD = 0.98) product was higher than that of low-priced (Mean = 3.40, SD = 1.01) product; When participants were at the add-to-cart stage, the perceived quality difference between high-priced (Mean = 5.00, SD = 0.87) and low-priced (Mean = 2.90, SD = 1.18) product significantly increased. Therefore, H1 was supported.

**Table 4:** Means and MANOVA results.

		Perceived product quality	Perceived monetary sacrifice
Add-to-cart stage	Low price	2.90 (SD = 1.18)	1.81 (SD = 1.00)
	High price	5.00 (SD = 0.87)	3.92 (SD = 1.16)
Place-an-order stage	low price	3.40 (SD = 1.01)	1.61 (SD = 0.84)
	High price	4.89 (SD = 0.98)	4.28 (SD = 1.16)
Main effect of stage		*F* = 2.92, *p* = 0.09	*F* = 0.44, *p* = 0.51
Main effect of price		*F* = 250.26, *p* = 0.00	*F* = 415.14, *p* = 0.00
Interaction effect		*F* = 7.37, *p* = 0.01	*F* = 5.85, *p* = 0.02

The results showed that the main effect of the decision-making stage on perceived monetary sacrifice was not significant [*F*(1, 318) = 0.44, *p* = 0.51], while the main effect of the price was significant [*F*(1, 318) = 415.14, *p* = 0.00]. Compared with the low price (Mean = 1.71, SD = 0.93), the high price (Mean = 4.10, SD = 1.17) product was perceived to have higher monetary sacrifice. What’s more, the interaction effect between the decision-making stage and price was significant [*F*(1, 318) = 5.85, *p* = 0.02]. When participants were in the add-to-cart stage, the perceived monetary sacrifice of high-priced (Mean = 3.92, SD = 1.16) product was higher than that of low-priced (Mean = 1.81, SD = 1.00) product; When participants were at the place-an-order stage, the perceived difference in monetary sacrifice between high-priced (Mean = 4.28, SD = 1.16) and low-priced (Mean = 1.61, SD = 0.84) products significantly increased. Therefore, H2 was supported.

We then conducted additional analyses to exclude alternative possible explanations. Firstly, we conducted ANOVA on participants’ experience, familiarity, knowledge of backpacks, product image attractiveness, involvement, and mood in the experiment. The results showed that, except for the main effect of decision-making stage on mood [*F*(1, 318) = 8.62, *p* = 0.00], there was no significant difference between the four groups of subjects in these perceptions. Secondly, the OLS regression results indicated that perceived product quality was not significantly associated with participants’ usage experience (*β* = −0.00, *p* = 0.98), knowledge (β = −0.05, *p* = 0.45), product image attractiveness (β = −0.05, *p* = 0.38), involvement (β = −0.05, *p* = 0.54), or mood (β = 0.06, *p* = 0.49), but was significantly associated with familiarity (β = 0.15, *p* = 0.03). Perceived monetary sacrifice was not significantly associated with participants’ usage experience (β = − 0.03, *p* = 0.60), familiarity (β = −0.01, *p* = 0.92), knowledge (β = 0.06, *p* = 0.41), product image attractiveness (β = −0.07, *p* = 0.27), involvement (β = 0.07, *p* = 0.45), or mood (β = −0.06, *p* = 0.56). Therefore, we excluded the possible explanation that participants’ different perceptions of product quality and monetary sacrifice were induced by their levels of usage experience, knowledge, attractiveness of backpack images, level of involvement, or mood. Taken the above two tests together, although there were significant differences in participants’ mood during the different decision-making stages, mood was not significantly associated with perceived product quality or monetary sacrifice, which ruled out the possible effect of mood. Similarly, although there was a significant correlation between participants’ familiarity with backpacks and perceived product quality, there was no significant difference in familiarity among different groups of participants, which thus excluded the possible effect of familiarity. Thirdly, we used gender variables as independent variables in the MANOVA. The results showed that there were no interaction effects between gender and decision-making stage or price, and gender did not have a significant main effect. Therefore, we excluded the potential impact of gender differences on the experimental results. Finally, we included all control variables (i.e., participants’ experience, familiarity, knowledge, backpack image attractiveness, involvement, mood, and age) in MANOVA. The results remained robust.

#### Effects on attitudes toward product

OLS regression analysis was performed by using perceived product quality and perceived monetary sacrifice as independent variables, and attitudes toward the product as the dependent variable. The results indicated that perceived product quality (β = 0.74, *p* = 0.00) and perceived monetary sacrifice (β = −0.11, *p* = 0.01) had a significant impact on participants’ product attitudes (R-squared = 0.49). In addition, when all control variables (i.e., gender, age, usage experience, familiarity, knowledge, backpack image attractiveness, involvement, and mood) were included in the regression, the results remained robust (R-squared = 0.52).

#### Conditional effects on attitudes toward product

Based on the estimation method proposed by Hayes (Hayes, 2017), we conducted a PROCESS analysis to examine the mediating roles of perceived product quality and perceived monetary sacrifice. The results of direct effects indicated that, when participants were at the add-to-cart stage, the direct effect of price on product attitude was significant (direct effect = 0.70, 95% Bootstrap CI = [0.30, 1.10]), whereas when participants were in the place-an-order stage, the direct effect was not significant (95% Bootstrap CI = [−0.14, 0.68]; see in Table 5). The indirect effects mediated by perceived product quality and perceived monetary sacrifice are shown in Table 5. The bootstrapping results revealed that the mediating effects of both perceived product quality (95% confidence interval with 5,000 bootstrap samples is [−0.70, −0.12]) and perceived monetary sacrifice (95% confidence interval with 5,000 bootstrap samples is [−0.24, −0.01]) were significantly moderated by decision-making stage.

**Table 5:** Results of PROCESS analyses.

		Effect	95% Bootstrap CI
Direct effect	Add-to-cart Stage	0.70	[0.30, 1.10]
	Place-an-order Stage	0.27	[−0.14, 0.68]
Indirect effect: perceived product quality	Moderated Mediation	−0.40	[−0.70, −0.12]
	Add-to-cart stage	1.38	[1.05, 1.73]
	Place-an-order stage	0.98	[0.68, 1.28]
Indirect effect: perceived monetary sacrifice	Moderated mediation	−0.10	[−0.24, −0.01]
	Add-to-cart stage	−0.37	[−0.68, −0.10]
	Place-an-order stage	−0.47	[−0.86, −0.13]

Specifically, compared with the place-an-order stage (95% confidence interval with 5,000 bootstrap samples is [0.68, 1.28]), the indirect effect of product price at the add-to-cart stage (95% confidence interval with 5,000 bootstrap samples is [1.05, 1.73]) on product attitude was stronger mediated by perceived quality. That is, compared with the place-an-order stage (Mean low-price = 3.94, SD low-price = 1.19; Mean high-price = 4.71, SD high-price = 1.14), higher product price at the add-to-cart stage (Mean low-price = 3.23, SD low-price = 1.30; Mean high-price = 4.93, SD high-price = 0.95) could induce more positive product attitude by improving perceived product quality. Thus, H3 was supported.

In contrast, compared with the add-to-cart stage (95% confidence interval with 5,000 bootstrap samples is [−0.68, −0.10]), the indirect effect of product price at the place-an-order stage (95% confidence interval with 5,000 bootstrap samples is [−0.86, −0.13]) on product attitude was stronger mediated by perceived monetary sacrifice. That is, compared with the add-to-cart stage (Mean low-price = 3.23, SD low-price = 1.30; Mean high-price = 4.93, SD high-price = 0.95), higher product price at the place-an-order stage (Mean low-price = 3.94, SD low-price = 1.19; Mean high-price = 4.71, SD high-price = 1.14) could induce less positive product attitude by decreasing perceived monetary sacrifice. Thus, H4 was supported.

## Discussion

Our study investigates the interplay effects of decision-making stage and product price on attitudes toward the product and the mediating roles of perceived product quality and monetary sacrifice. The results indicate that consumers at different decision-making stages (i.e., add-to-cart and place-an-order) tend to pay attention to different aspects of product price perception, resulting in different product attitudes. Specifically, we verified that consumers tend to focus on the quality perception conveyed by price information at the add-to-cart stage, whereas they tend to focus on the monetary sacrifice perception conveyed by price information at the place-an-order stage. By conducting a behavioral experiment, we provide strong evidence to prove the differences in the effects of price on perceived product quality and monetary sacrifice, and demonstrate that this difference leads to different product attitudes among consumers at the add-to-cart and place-an-order stages.

Specifically, our study demonstrates that the positive effect of price on consumers’ perception of product quality is significantly stronger at add-to-cart stage than that at place-an-order stage. In contrast, the positive effect of price on consumers’ perception of product quality is significantly weaker at add-to-cart stage than that at place-an-order stage. Furthermore, our findings show that these differences further induce different attitudes toward products at add-to-cart stage and place-an-order stage. That is, consumers’ attitude toward product at add-to-cart stage is significantly more positive that at place-an-order stage because of the strengthened effect of price on perception of product quality. While consumers’ attitude toward product at place-an-order stage is significantly less positive than that at add-to-cart stage because of the strengthened effect of price on perception of monetary sacrifice. Therefore, all our hypotheses are supported.

### Theoretical contributions

Our findings make several important theoretical contributions. Firstly, our study offers new insights into the virtual shopping cart literature by investigating how product price affects consumer perceptions and product attitude at decision-making stages before and after cart use. On the one hand, previous literature mainly focused on exploring the influencing factors of consumer perceptions and attitudes at add-to-cart stage or place-an-order stage (Boyle et al., 2018; Cho et al., 2006; Huang et al., 2018; Moore and Mathews, 2008). Very few efforts have been taken to investigate the influence of product information. Our findings show that higher product price elicit consumers’ perception of product quality at the add-to-cart stage and thus lead to more positive product attitude, whereas a lower product price effectively decreases consumers’ perception of monetary sacrifice and further contributes to a more positive attitude toward the product at the place-an-order stage. On the other hand, the extant literature exploring influencing factors at the add-to-cart or place-an-order stages mainly regards place-an-order behavior as a natural consequence of add-to-cart behavior, but does not account for the essential fact that the promotion effect of these factors at the add-to-cart stage may not necessarily exist in the place-an-order stage (Tang and Lin, 2019). To the best of our knowledge, we are among the first to examine how the same piece of product information (i.e., price) at the add-to-cart stage affects consumer perceptions and attitudes differently at the place-an-order stage. Specifically, our findings demonstrate that product information (i.e., higher prices) that can promote product quality perception and product attitude at the add-to-cart stage may not be such effective at the place-an-order stage, whereas lower prices that improve attitudes toward products at the place-an-order stage may not be such beneficial for consumer attitude at the add-to-cart stage.

Secondly, this study extends the understanding of the impact of product price at different decision-making stages. Prior studies on price have explored the impact of factors related to product traits (Shirai, 2015; Völckner et al., 2012), consumer traits (Park et al., 2020; Yang et al., 2019; Choi et al., 2020), decision-making contexts (Jeong et al., 2019; Joo et al., 2020; Wakefield and Wakefield, 2018), and information frameworks (Allard and Griffin, 2013; Allard and Griffin, 2017; Kim et al., 2019) on how price elicits product quality perception and cost perception. However, very few efforts have been allocated to focus on the potential effects of the decision-making stages. Our study complements existing research by demonstrating that the effect of price on the perception of product quality is more prominent in the add-to-cart stage, resulting in a more positive effect of price on attitudes toward a product. In contrast, consumers are more concerned about the perceptions of monetary sacrifice brought about by price at the place-an-order stage, resulting in a less positive effect of price on product attitude.

Thirdly, our study contributes to the application of consumer shopping goal stage theory by investigating how the same piece of product information helps consumers facilitate decision-making process at different stages. Previous research mainly leveraged this theory to examine how to target different information at specific stages to facilitate the decision-making process (Luo et al., 2019; Song et al., 2017; Trzebinski et al., 2023). Seldom has any study examined how targeting the same piece of information can facilitate decision-making process. Our study adds to the literature by providing evidence on how price information can effectively help consumers form product attitudes at different stages by emphasizing different perceptions of price (i.e., perceived product quality and monetary sacrifice). Thus, our findings highlight the importance of taking multi-roles of product information in consumer perceptions into consideration when applying the consumer shopping goal stage theory to investigate the effect of information targeting strategies.

### Practical implications

Our study also has several practical implications. Firstly, this study provides specific marketing guidelines for e-commerce retailers to utilize price information. This study shows that consumers rely more on price information to make quality judgments when they are in the add-to-cart stage (compared to the place-an-order stage). Therefore, it is recommended that different aspects of price information be emphasized at different decision-making stages to promote consumers’ perceptions of product quality and attitude. For example, e-retailers can emphasize high-quality inferences about product information to attract consumers who have not yet added products to their shopping carts. When consumers select products from shopping cart lists, e-retailers are encouraged to emphasize a lower monetary sacrifice of products to attract consumers to place orders.

Secondly, this study highlights the different roles product price playing in different decision-making stages. This study shows that e-retailers should utilize price information differently when they aim to attract consumers in the add-to-cart stage compared to the place-an-order stage. For example, when e-retailers want to attract consumers to add products to their shopping carts, they can emphasize the relatively high original price of the product to show its high quality. When they want to attract consumers to directly place an order, they can highlight the relatively low promotional price of the product to reduce consumers’ perception of monetary sacrifice.

Thirdly, this study identifies the key factors (i.e., perceived quality and monetary sacrifice) that account for consumers’ attitudes toward products at different decision-making stages. Our findings indicate that consumers tend to make quality inferences from product information at the add-to-cart stage, while paying more attention to monetary sacrifice at the place-an-order stage. Therefore, managers of e-commerce platforms and online retailers should design marketing strategies based on consumers’ psychological demands at various stages of decision-making. For example, e-retailers should place greater emphasis on the excellent quality and performance of products when providing advertisements to consumers who have not yet added products to their shopping carts. For products already in shopping cart lists, it is recommended to emphasize product warranty services and after-sales guarantees to minimize consumer concerns about monetary sacrifice.

### Limitations and research directions

Our research is also subject to several limitations. Firstly, our study adopted backpacks as the target product, which have been widely adopted in previous literature and are commonly used by both female and male college students. Considering that prior studies have also pointed out that product types may have a boundary effect on consumer perceptions (Park and Lee, 2009; Park and Moon, 2010), future research is recommended to utilize more types of products and see if our conclusions still hold, or further incorporate product types as moderators into the theoretical research model.

Secondly, our study focuses on how product prices induce consumer perceptions and attitudes at different decision-making stages. Considering that there are many types of product information in online purchasing, we recommend future research to continue our effort by focusing on other types of product information and exploring the interplay effect of product information and consumers’ decision-making stages.

Thirdly, our study relies on behavioral experiment to investigate the effects of product prices and decision-making stages. Although behavioral experiment is more suitable for our research goal, we suggest future studies to test our research hypotheses in real e-commerce settings, such as field experiments and natural experiments.

## Data Availability

The raw data supporting the conclusions of this article will be made available by the authors, without undue reservation.
